# A survey of Canadian intensivists' resuscitation practices in early septic shock

**DOI:** 10.1186/cc5962

**Published:** 2007-07-10

**Authors:** Lauralyn A McIntyre, Paul C Hébert, Dean Fergusson, Deborah J Cook, Ashique Aziz, the Canadian Critical Care Trials Group

**Affiliations:** 1 University of Ottawa Centre for Transfusion and Critical Care Research, Clinical Epidemiology Unit of the Ottawa Hospital, Ottawa Health Research Institute, 501 Smyth Rd Ottawa, Ontario, Canada K1H 8L6; 2 Ottawa Health Research Institute, Clinical Epidemiology Program of the Ottawa Hospital, 501 Smyth Rd, Ottawa, Ontario, Canada, K1H 8L6; 3 Clarity Research Group, Department of Medicine and Clinical Epidemiology & Biostatistics, McMaster University Health Sciences Centre, 1200 Main Street West, Hamilton, Ontario, Canada L8N 3Z5; 4 Ottawa Health Research Institute, University of Ottawa, Ottawa, Ontario, Canada

## Abstract

**Introduction:**

Recent evidence suggests that early, aggressive resuscitation in patients with septic shock reduces mortality. The objective of this survey was to characterize reported resuscitation practices of Canadian physicians caring for adult critically ill patients with early septic shock.

**Methods:**

A scenario-based self-administered national survey was sent out to Canadian critical care physicians. One hypothetical scenario was developed to obtain information on several aspects of resuscitation in early septic shock, including monitoring and resuscitation end-points, fluid administration, red blood cell transfusion triggers, and use of inotropes. The sampling frame was physician members of Canadian national and provincial critical care societies.

**Results:**

The survey response rate was 232 out of 355 (65.3%). Medicine was the most common primary specialty (60.0%), most respondents had practiced for 6 to 10 years (30.0%), and 82.0% were male. The following monitoring devices/parameters were reported as used/measured 'often' or 'always' by at least 89% of respondents: oxygen saturation (100%), Foley catheters (100%), arterial blood pressure lines (96.6%), telemetry (94.3%), and central venous pressure (89.2%). Continuous monitoring of central venous oxygen saturation was employed 'often' or 'always' by 9.8% of respondents. The two most commonly cited resuscitation end-points were urine output (96.5%) and blood pressure (91.8%). Over half of respondents used normal saline (84.5%), Ringers lactate (52.2%), and pentastarch (51.3%) 'often' or 'always' for early fluid resuscitation. In contrast, 5% and 25% albumin solutions were cited as used 'often' or 'always' by 3.9% and 1.3% of respondents, respectively. Compared with internists, surgeons and anesthesiologists (odds ratio (95% confidence interval): 9.8 (2.9 to 32.7) and 3.8 (1.7 to 8.7), respectively) reported greater use of Ringers lactate. In the setting of a low central venous oxygen saturation, 52.5% of respondents reported use of inotropic support 'often' or 'always'. Only 7.6% of physicians stated they would use a red blood cell transfusion trigger of 100 g/l to optimize oxygen delivery further.

**Conclusion:**

Our survey results suggest that there is substantial practice variation in the resuscitation of adult patients with early septic shock. More randomized trials are needed to determine the optimal approach.

## Introduction

Severe sepsis accounts for approximately 3% of admissions to hospital and 10% of admissions to the intensive care unit (ICU), and it is the 10th leading cause of death in the ICU [1,2]. Despite decades of intense therapeutic investigation, the mortality from severe sepsis and septic shock remains between 30% and 60% [3,4].

Aggressive resuscitation is the cornerstone of early treatment for patients with severe sepsis and septic shock [5]. In a landmark randomized controlled trial of goal-directed therapy in early septic shock, hospital mortality in the goal-directed group was reduced by 17% [6]. Both standard therapy and goal-directed therapy groups received algorithm driven care, with resuscitation end-points including mean arterial pressure, central venous pressure, and urine output as goals. However, an additional resuscitation end-point for the goal-directed resuscitation group was to achieve central venous oxygen saturation (ScvO_2_) of 70% or greater; this resuscitation end-point resulted in greater use of dobutamine, red blood cell (RBC) transfusions, and significant amounts of crystalloid and colloid fluid during the first 6 hours of care [6]. Given the many different interventions in algorithm driven care, it is unclear which aspect of the goal-directed intervention influenced survival most.

To elucidate self-reported resuscitation interventions and describe which aspects of goal-directed therapy are used by Canadian ICU physicians, we conducted a national survey of early adult septic shock resuscitation management.

## Materials and methods

### Study participants

A self-administered survey was sent to Canadian critical care physicians identified using national and provincial critical care society mailing lists. The lists were verified and supplemented by contacting all major critical care program directors in each province. We merged lists and de-duplicated names, and identified 489 potentially eligible physicians. We then excluded fellows, retired members, physicians practicing outside Canada, pediatric intensivists, and physicians with no forwarding address. In total, 355 critical care practitioners were ultimately considered eligible and were mailed the survey between January 2004 and May 2004.

### Survey development

The scenario and corresponding questions were developed through an iterative process among the investigative team, and in consultation with members of the Canadian Critical Care Trials Group, representing 140 critical care clinicians from across the country. One scenario was chosen to represent a typical patient with septic shock, which also enabled survey completion within 10 min to minimize respondent burden.

The scenario described a 55-year-old woman in the emergency room with vital signs compatible with septic shock after a 1 l bolus of normal saline. We described an older patient to reflect the commonest age profile of this population, and because older age is associated with increased mortality from septic shock [2] (Additional file 1). Three questions were asked to elucidate usual monitoring parameters, volume resuscitation end-points, and resuscitation fluid preferences. We then altered the scenario to reflect the same patient but with optimized intravascular volume and blood pressure, reduced metabolic demand, and inadequate oxygen delivery manifested by a low ScvO_2_. We used noradrenaline (norepinephrine) in the scenario because it is often considered a first-line vasopressor agent for use in septic shock [7]. The patient was mechanically ventilated with sedation and analgesia to represent a situation in which metabolic demand had been minimized. We then asked whether physicians would intervene with RBCs and inotropic agents in response to a low ScvO_2_. The final version of the survey included one scenario with five questions eliciting information on resuscitation end-points and interventions (Additional file 1). A 5-point Likert scale (never, rarely, sometimes, often, always) was used to elicit answers about preferred monitoring parameters, volume resuscitation end-points, resuscitation fluid, and inotropes. For the RBC transfusion trigger question, we divided the hemoglobin level into seven distinct thresholds (60, 70, 80, 90, 100, 110, and 120 g/l), because previous surveys demonstrated that 95% of physicians chose transfusion thresholds for the critically ill that were consistent with the ones in our survey [8,9].

We also recorded information on physician and institution characteristics, including age, sex, primary specialty (medicine, surgery, anesthesia, or other), years in practice (0 to 5, 6 to 10, 11 to 15, or >15), number of weeks worked in the ICU (0 to 10, 11 to 20, or >20), and academic affiliation (university or community hospital).

### Survey preparation

The scenario was assessed for content, clarity, and realism by 17 members of the Canadian Critical Care Trials Group who piloted the survey. The survey was translated into French for physicians who lived in Quebec, Canada. The Research Ethics Committee of the Ottawa Hospital approved this study.

### Survey administration

We mailed the survey with a pre-stamped envelope. Physicians who had not yet returned their forms received a reminder postcard 4 to 6 weeks after the first mailing. After another 4 to 6 weeks, nonrespondents were sent a second survey.

### Statistical analysis

We described physician and institution characteristics (age, sex, primary specialty, years in practice, weeks worked in ICU, and academic affiliation) as well as the different resuscitation interventions (normal saline, Ringers lactate, pentastarch, RBC transfusion triggers, inotropes) using proportions. All resuscitation intervention responses were dichotomized into often/always versus sometimes/rarely/never. Reported monitoring parameters and volume resuscitation end-points were graphically represented by using a compressed 5-point Likert scale (often/always, sometimes, and rarely/never).

To examine practice variation regarding resuscitation intervention variables, we conducted multivariable logistic regression analyses. The dependent variables included all resuscitation interventions (normal saline, Ringers lactate, pentastarch, RBCs, and inotropes, dichotomized into always/often versus sometimes/rarely/never). Independent variables were forced into the models and included all ICU physician characteristics (age (increasing increments of 10 years), sex, primary specialty (medicine, surgery, anesthesia, other), years in practice (0 to 5, 6 to 10, 11 to 15, >15 years), and weeks worked in the ICU (0 to 10, 11 to 20, >20)). We expressed associations identified in the multivariable analyses as odds ratios (ORs) and 95% confidence intervals (CIs). An OR of less than 1 was associated with less frequent use of the resuscitation intervention, and an OR of greater than 1 was associated with more frequent use.

## Results

### Survey respondents

We identified a total of 489 potential respondents. From this list, 134 were deemed ineligible for the following reasons: they did not primarily practice critical care (*n *= 50), they did not treat adults in their practice (*n *= 23); they were retired (*n *= 4); or their address was unknown (*n *= 57). A total of 232 of 355 eligible respondents replied (response rate 65.3%). The physicians who responded mostly specialized in medicine (60.0%), had been practicing for 6 to 10 years (30.0%), and were primarily male (82.0%; Table 1).

**Table 1:** Physician characteristics

Physician characteristics	Percentage
Age mean (SD)	46.4 (7.3)
Sex (male)	82.0
Primary specialty	
Medicine	60.0
Surgery	14.8
Anesthesia	22.6
Other	2.6
Number of years in practice	
0 to 5	22.4
6 to 10	30.0
11 to 15	19.3
>15	28.3
Number weeks worked in ICU	
0 to 10	74.0
11 to 20	58.7
>20	18.3
Academic affiliation	
University	74.1
Community	25.9

There were 232 respondents in total. ICU, intensive care unit; SD, standard deviation.

### Resuscitation monitors and end-points

**Figure 1:**
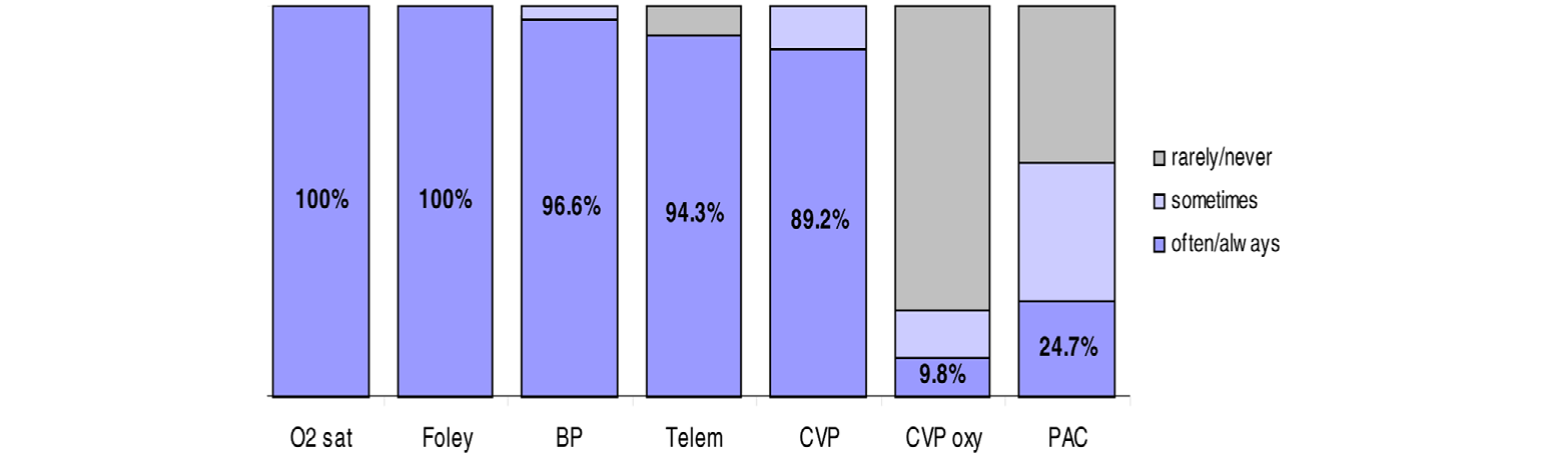
Monitoring parameters used by ICU physicians. BP, intra-arterial blood pressure; CVP, central venous pressure; CVP oxy, continuous monitoring of central venous oxygen saturation; Foley, Foley catheter; ICU, intensive care unit; O2 sat, oxygen saturation; PAC, pulmonary artery catheter; Telem, telemetry.

**Figure 2:**
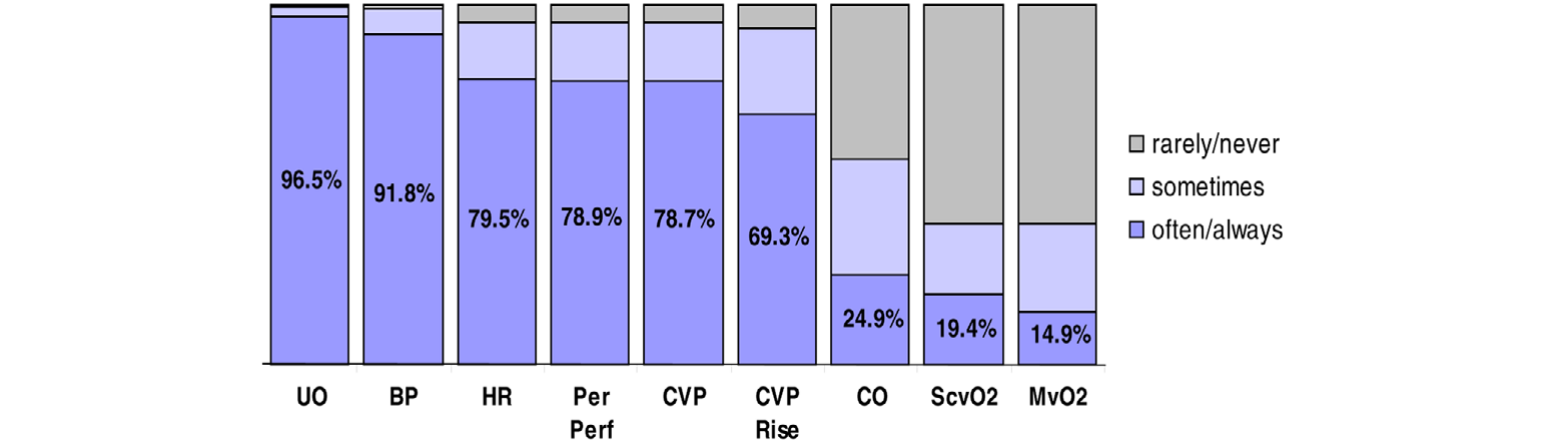
ICU physicians stated volume resuscitation end-points. BP, blood pressure; CO, cardiac output; CVP, central venous pressure; CVP rise, sustained rise in central venous pressure; HR, heart rate; ICU, intensive care unit; MvO2, mixed venous oxygen saturation; Per Perf, peripheral perfusion; ScvO2, central venous oxygen saturation; UO, urine output.

The following monitoring devices/parameters were reportedly used 'often' or 'always' by at least 89% of respondents to monitor early septic shock: oxygen saturation (100%), foley catheters (100%), arterial blood pressure lines (96.6%), telemetry (electrocardiographic monitoring; 94.3%), and central venous pressure (89.2%; Figure 1). The pulmonary artery catheter and continuous monitoring of ScvO_2 _were used 'often' or 'always' by 24.7% and 9.8% of respondents, respectively. ICU physicians reported use of several physiologic measures (resuscitation end-points) 'often' or 'always' to evaluate whether a patient was adequately volume resuscitated in the early phases of septic shock (Figure 2). Urine output and blood pressure were reported as used 'often' or 'always' most frequently (96.5% and 91.8%, respectively), followed by heart rate (79.5%), peripheral perfusion (78.9%), central venous pressure (78.7%), and a sustained rise in central venous pressure in association with a fluid challenge (69.3%). Of respondents, 19.4% reported use of ScvO_2 _as a volume resuscitation end-point 'often' or 'always'.

### Resuscitation interventions

Normal saline, Ringers lactate, and pentastarch were reported as used 'often' or 'always' by 84.5%, 52.2%, and 51.3% of respondents, respectively, as resuscitation fluids of choice for early septic shock (Table 2). Use of 5% and 25% albumin was less common (3.9% and 1.3% or respondents, respectively). The combination of normal saline, Ringers lactate, and pentastarch were reported as used 'often' or 'always' by 21.9% of physicians; 5.2% stated that they used normal saline alone; 0.5% stated that they used Ringers lactate alone; and 5.7% stated that they used crystalloid fluid alone (normal saline and Ringers lactate). No physicians stated that they would use pentastarch alone 'often' or 'always' as their resuscitation fluid.

**Table 2:** Resuscitation interventions

Resuscitation interventions	Percentage
Fluid intervention	
Normal saline	84.5
Ringer's lactate	52.2
Pentastarch	51.3
5% albumin	3.9
25% albumin	1.3
Fluid intervention combinations	
Normal saline + ringers lactate + pentastarch	21.9
Normal saline + ringers lactate only	5.7
Normal saline only	5.2
Ringers lactate only	0.5
Pentastarch only	0
Red blood cell transfusion trigger (g/l)	
60	2.2
70	42.2
80	32.4
90	15.1
100	7.6
110	0.4
120	0
Inotropes	
Never	7.2
Rarely	13.6
Sometimes	26.7
Often	42.1
Always	10.4

There were 232 respondents in total. The percentages reflect an 'often' or 'always' response to questions regarding the resuscitation interventions.

Only 7.6% of ICU physicians reported that they would transfuse RBCs at a hemoglobin trigger of 100 g/l if the ScvO_2 _was 50% in a patient who had reduced metabolic demand and optimized intravascular volume and blood pressure. However, 76.8% of physicians stated that they would use a hemoglobin transfusion trigger of 80 g/l or less. Of physicians, 52.5% stated that they would use inotropes 'often' or 'always' if the ScvO_2 _remained below the set goal after volume resuscitation and blood pressure optimization, minimization of metabolic demand, and administration of RBCs to improve oxygen delivery (Table 2).

### Influence of physician characteristics on responses

**Figure 3:**
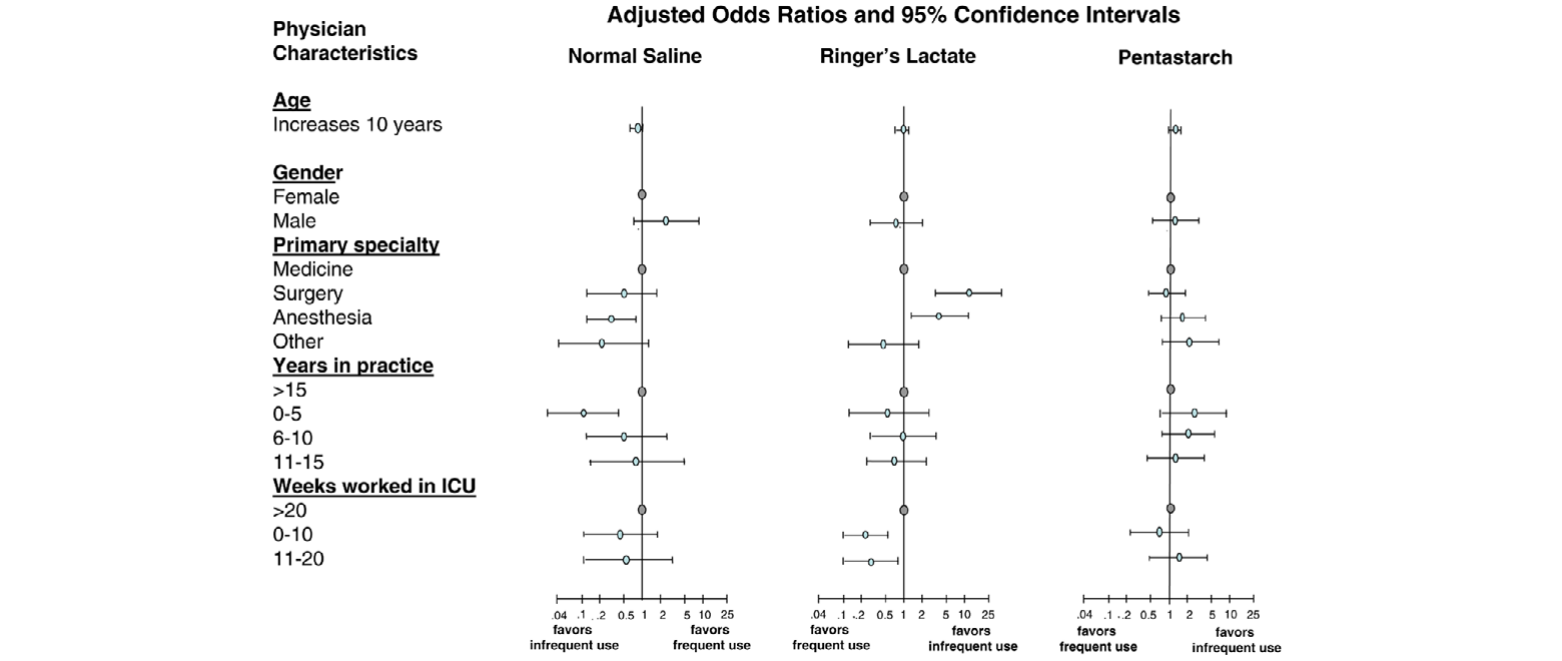
Association between physician characteristics and resuscitation fluids. The figure shows the association between resuscitation fluid preferences and intensive care unit (ICU) physician characteristics using multivariable logistic regression analyses. An odds ratio below 1 reflects less frequent use (sometimes/rarely/never) of the resuscitation intervention. An odds ratio greater than 1 reflects increased frequent use (always/often) of the resuscitation intervention.

**Figure 4:**
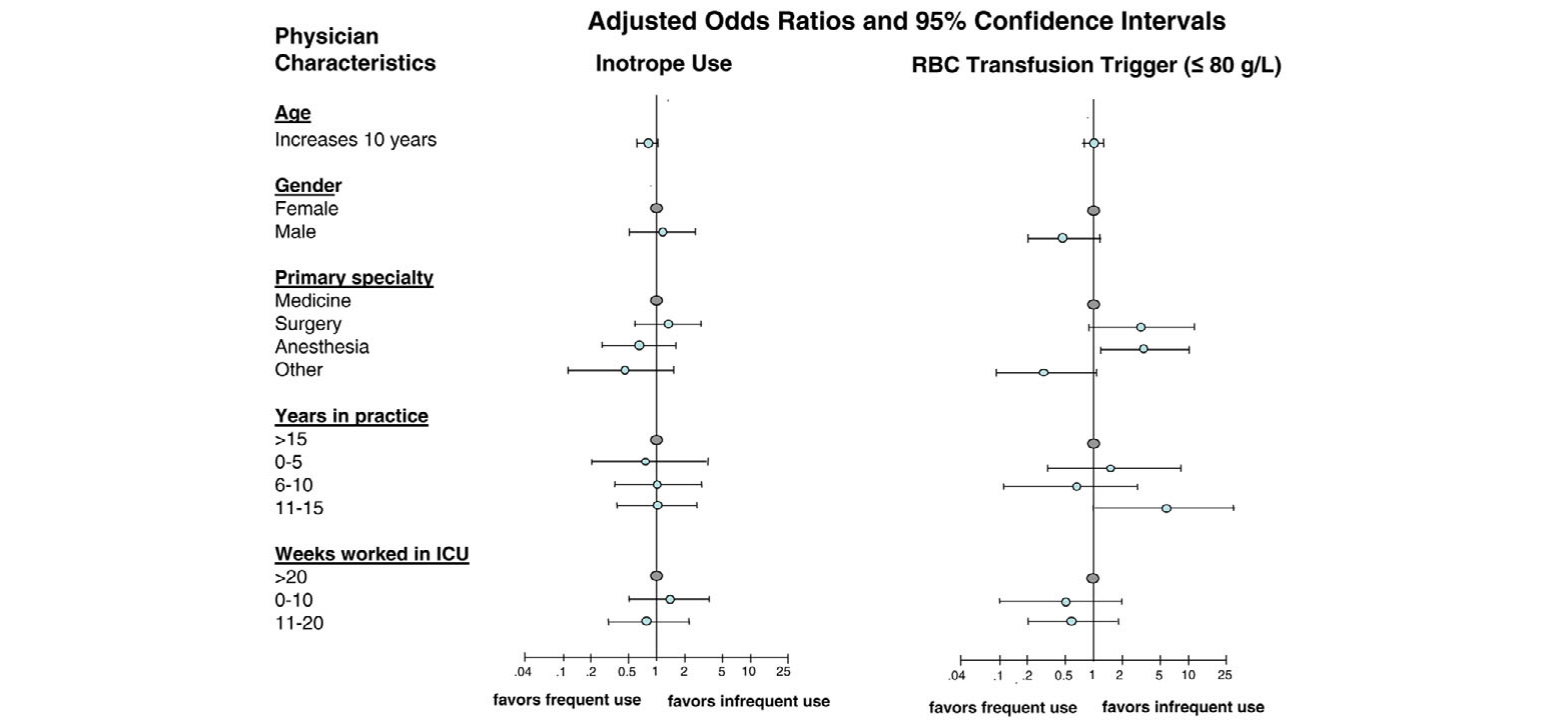
Association between physician characteristics and resuscitation interventions. The figure shows the association between resuscitation intervention preferences and intensive care unit (ICU) physician characteristics using multivariable logistic regression analyses. An odds ratio below 1 reflects less frequent use (sometimes/rarely/never) of the resuscitation intervention. An odds ratio above 1 reflects increased frequent use (always/often) of the resuscitation intervention. RBC, red blood cell.

Using multivariable analyses, we also examined whether different physician characteristics (age, sex, primary specialty, years in practice, weeks worked in ICU) were associated with differential use of fluids, RBCs, and inotropes. Anesthesiologists (OR 3.8, 95% CI 1.7 to 8.7) and surgeons (OR 9.8, 95% CI 2.9 to 32.7), compared with internists, reported greater use of Ringers lactate (Figure 3). Physicians who spent less time working in the ICU reported lower use of Ringers lactate as compared with those who spent 20 weeks per year or more working in the ICU (0 to 10 weeks: OR 0.2, 95% CI 0.1 to 0.7; 11 to 20 weeks: OR 0.4, 95% CI 0.1 to 0.9; Figure 3). Anesthesiologists were more likely than internists to report using a RBC transfusion trigger of 80 g/l or less (OR 3.9, 95% CI 1.2 to 12.9; Figure 4). No associations were detected between physician characteristics and use of pentastarch or inotropic agents (Figures 3 and 4, respectively).

## Discussion

The results of our survey suggest that Canadian ICU physicians commonly use crystalloid fluids such as normal saline and ringers lactate, and the colloidal fluid pentastarch for early septic shock resuscitation; use of albumin is reportedly much less frequent. Blood pressure and urine output were cited as the two most common volume resuscitation end-points. Among different specialties, physicians also appear to have divergent fluid resuscitation preferences; indeed, anesthesiologists and surgeons reported greater use of Ringers lactate than did internists. Compared with internists, anesthesiologists also more frequently reported using a low hemoglobin transfusion trigger of 80 g/l or less.

Interestingly, only 10% of Canadian ICU physicians stated that they would use continuous measurements of ScvO_2 _even if this monitoring parameter was available for early septic shock. However, 53% said that they would use intropic agents, and all physicians stated they would transfuse patients in response to a low ScvO_2_. These responses suggest that although physicians may infrequently use continuous monitoring of ScvO_2_, they may use it intermittently or in some patients to help guide therapy. We conclude that the protocol presented by Rivers and coworkers [6] for early septic shock resuscitation has been variably adopted by Canadian ICU physicians, perhaps for several reasons.

Although it was a well conducted landmark trial in goal-directed resuscitation, supported by the Surviving Sepsis Campaign Guidelines for management of severe sepsis and septic shock [10], it was a single-center study and has not yet been replicated. Although some centers have evaluated and adopted this early goal-directed resuscitation protocol as part of their clinical practice [11-15], many questions remain. The benefit seen in the early goal-directed group may have been due to expedient resuscitation rather than continuous monitoring of ScvO_2 _itself [16,17]. Indeed, one explanation for the negative results of the goal-directed resuscitation trial conducted by Gattinoni and coworkers [18], which incorporated ScvO_2 _as a resuscitation end-point, as compared with the trial reported by Rivers and coworkers could be the time to initiate goal-directed therapy. Rivers and colleagues randomized patients into the study within 1 hour of arrival in the emergency room, whereas Gattinoni and coworkers enrolled patients within 48 hours of admission to the ICU [17,18]. Furthermore, it is unclear whether intermittent as compared with continuous ScvO_2 _monitoring is sufficient to detect low ScvO_2 _in early septic shock. Another reason for reported low adoption of continuous monitoring of ScvO_2 _may relate to lack of resources. In a survey of 30 academic emergency room physicians from the USA, only 7% reported use of early goal-directed therapy in the emergency room. Major reported barriers for implementation of early goal-directed therapy included the need for specialty monitoring equipment (75%), amount of resources needed (43%), the need for central venous catheter cannulation (36%), and too much emergency physician time required (29%) [19].

The use of RBCs to augment oxygen delivery when the ScvO_2 _was below 70% was a controversial aspect of the Rivers resuscitation algorithms. Results of our survey suggested that only 7% of physicians would transfuse at a hemoglobin of 100 g/l, and 75% stated they would transfuse at a target hemoglobin that was as low as 80 g/l or less. An important reason for accepting a lower RBC transfusion trigger in the critically ill patient population probably relates to evidence from the Canadian led Transfusion in Critically Ill (TRICC) trial [20], which demonstrated a lower transfusion trigger (hemoglobin 70 g/l) was just as effective as a more conservative transfusion trigger (hemoglobin 90 g/l). However, results from the TRICC trial may not apply to patients with early septic shock, because patients with sepsis represented only 5% of the TRICC patient population [21]. Furthermore, the study by Rivers and coworkers did not specifically address the question of an optimal RBC transfusion trigger in this setting, and hence it is difficult to know whether this liberal transfusion trigger was responsible for the mortality benefit seen in the goal-directed group.

Half of our survey respondents indicated that they would start an inotropic agent in response to a low ScvO_2_. Lack of a uniform response may be related to concerns that use of these agents could potentially worsen myocardial oxygen consumption and cardiac arrhythmias in the setting of a heart that is already in high demand [22,23]. Furthermore, because the study by Rivers and coworkers was not specifically designed to test whether an inotropic agent improved outcome, it is difficult to know whether the contribution of inotropes, among many other components of the protocol in the intervention arm, was primarily responsible for the mortality benefit seen in the goal-directed group.

We found heterogeneous responses with regard to choice of resuscitation fluids in early septic shock. Although normal saline, Ringers lactate, and the colloid pentastarch were used at least 50% of the time, the use of either 5% or 25% albumin was infrequent. Reasons for minimal use of albumin may be related to the findings of a previous meta-analysis that suggested that albumin was not beneficial and was potentially harmful [24]. However, results from the Saline versus 4% Albumin Fluid Evaluation (SAFE) study has put some of this controversy to rest [25]. That study was a randomized controlled trial of 6,997 heterogeneous critically ill patients in need of volume resuscitation, which compared 4% albumin with normal saline. It found no difference in 28-day mortality between the two groups, but there was a trend toward lower mortality in a subgroup of patients with severe sepsis who received albumin (relative risk ratio of 0.87, 95% CI 0.74 to1.02). The results of this subgroup analysis have served to fuel the debate with regard to superiority of colloidal as compared with crystalloid fluid resuscitation in the setting of severe sepsis and septic shock, and call for a large prospective, randomized trial to confirm or refute this hypothesis.

Our survey examined stated adult ICU physician resuscitation practices within the context of early septic shock. There are four previously published critical care surveys. Two reflect European [26,27] and two reflect Canadian [28] and USA resuscitation practices [19]. The Canadian and European surveys focused specifically on choice of resuscitation fluids and rationale for these choices in heterogeneous critically ill patient populations. The US survey reported on the use and barriers to implementation of early goal-directed therapy [19], as compared with our survey, which elicited stated practices for both resuscitation monitoring techniques and numerous therapeutic interventions.

There are several potential limitations to the methods used in this study, including response bias and response rate. For example, the use of a hypothetical scenario may have resulted in critical care physicians stating that they use resuscitation parameters similar to those described by Rivers and coworkers more often than in actual practice. It is also possible that one of our questions on the RBC transfusion trigger might have prompted reports of a more restrictive transfusion trigger because we, the survey investigators, had conducted the only major randomized trial in this field. It is difficult to predict how the 35% of individuals who did not respond may have answered the questions and influenced the results and interpretation of the findings. However, it is difficult to speculate whether the nonresponding physicians would be entirely homogeneous and sufficiently different in their answers, and thus impact on our overall results. The universal caveat for all surveys holds for this one as well; what physicians say they do may not reflect what they actually do.

This survey examined the very topical issue of resuscitation in septic shock, a condition associated with high mortality. We surveyed physicians with different base specialties in university affiliated and community hospitals. Although surveys of stated practice may not reflect actual practice [8,20], it is important to conduct surveys early in research programs designed to address vexing problems such as resuscitation strategies, because they provide essential background information for the design of these trials.

## Conclusion

Our survey identified extremely diverse practices, which suggest a bright future for resuscitation research. Well designed randomized controlled trials addressing specific resuscitation questions with clinically important end-points have the greatest potential to improve the care and outcomes in this vulnerable population.

## Key messages

▪ Our survey response rate was 232 out of 355 (65.3%).

▪ The following parameters/monitoring devices were reported as used 'often' or 'always' by at least 89% of respondents: oxygen saturation (100%), Foley catheters (100%), arterial blood pressure lines (96.6%), telemetry (94.3%), and central venous pressure (89.2%); continuous monitoring of ScvO_2 _was used 9.8% of the time.

▪ The two most commonly cited resuscitation end-points were urine output (96.5%) and blood pressure (91.8%).

▪ Over half of respondents used normal saline (84.5%), Ringers lactate (52.2%), and pentastarch (51.3%) 'often' or 'always' for early fluid resuscitation; in contrast, 5% and 25% albumin solutions were cited as used 'often' or 'always' by 3.9% and 1.3% of respondents, respectively.

▪ In the setting of a low ScvO_2_, 52.5% of respondents stated that they used inotropic support 'often' or 'always'; only 7.6% of physicians stated that they would use a RBC transfusion trigger of 100 g/l to optimize oxygen delivery further.

## Abbreviations

CI = confidence interval; ICU = intensive care unit; OR = odds ratio; RBC = red blood cell; ScvO_2 _= central venous oxygen saturation; TRICC = Transfusion in Critically Ill.

## Competing interests

In 2003, Edwards Life Sciences provided Dr LA McIntyre with an $8000 unrestricted educational grant to help perform a pilot randomized controlled trial that examined the role of fluids in the setting of early septic shock. However, Edwards Life Sciences has not provided any funding for this present study.

## Authors' contributions

LM was responsible for the conception and design of the study, analysis of data, manuscript drafting, and all manuscript revisions. DC, DF, and PH aided with study design, data analysis and drafting, as well as manuscript revisions. AA aided with data analysis and manuscript revision.

## Supplementary Material

Additional file 1 A Word document containing the septic shock resuscitation survey questions. Click here for file
