# Supplementary material for: A survey of Canadian intensivists' resuscitation practices in early septic shock
Source: Crit Care. 2007 Jul 10;11(4):R74. doi: 10.1186/cc5962 (PMC2206518; doi:10.1186/cc5962)
Supplement: Additional file 1 — A Word document containing the septic shock resuscitation survey questions. [file cc5962-S1.doc]

**Data File 1: Septic Shock Resuscitation Survey Questions**

**Imagine you have been asked to see a 55 year old female that arrived in the emergency room with suspected septic shock. She is confused, with a blood pressure of 70/30, heart rate 135 beats per minute, respiratory rate of 25 breaths per minute, temperature 39.5 degrees Celsius and oxygen saturation is 96% on 3 liters nasal prongs. She received a total of 1 liter of intravenous normal saline over 15 minutes.**

**#1.** In your ICU, please indicate how often you would use (if available) the following monitoring devices in the initial 6 hours of resuscitating the patient described in the above scenario.

(Never; Rarely; Sometimes; Often; Always)

a) Oxygen saturation e) Central venous pressure

b) Foley catheter f) Central venous pressure with continuous ScV0_2_

c) Telemetry g) Pulmonary artery catheter

d) Intra - arterial blood pressure

**#2.** For the patient described in the above scenario, what type of fluid would you administer in the first 6 hours of resuscitation? (Never; Rarely; Sometimes; Often; Always)

a) Normal Saline d) 25% albumin

b) Ringers Lactate e) Pentastarch

c) 5% Albumin f) Other (please specify)

**#3:** For the patient described in the above scenario, what end points would you use to evaluate if the patient is adequately volume resuscitated? (Never; Rarely; Sometimes; Often; Always)

a) Heart Rate f) Sustained rise in central venous pressure

b) Blood Pressure g) ScV02

c) Peripheral perfusion h) MV02

d) Urine Output i) Cardiac output/index

e) Central Venous Pressure j) Other (please specify)

**To answer the next questions, please consider that the patient is now volume resuscitated. Heart rate is 90 bpm, mean arterial pressure is 70 mm Hg on 10 mcg/min of norepinephrine and central venous pressure is 12 mm hg. She is now intubated, ventilated, and sedated on a midazolam and morphine infusion. A right internal jugular central venous catheter in good position shows a central venous oxygen saturation of 50% (assume the catheter is placed just above the right atrium). You are now 2 hours into your resuscitation.**

**#5.** For the patient described in the above scenario, what is the lowest hemoglobin trigger that you would use to administer a red blood cell transfusion?

**□ 60 g/L □ 70 g/L □ 80 g/L □ 90 g/L □ 100 g/L □ 110 g/L □ 120 g/L Other (please specify):**

**#6.** For the patient described in the above scenario, if the central venous saturation is still below your set goal after administration of red blood cell transfusion(s), please state how often you would start an inotropic agent to further increase oxygen delivery:

**□ Never □ Rarely □ Sometimes □ Often □ Always Other (please specify)**
